# Symptoms of Anxiety and Depression Among Adolescents Before vs During COVID-19–Related School Closures in China

**DOI:** 10.1001/jamanetworkopen.2022.41752

**Published:** 2022-11-14

**Authors:** Miao Qu, Kun Yang, Yujia Cao, Xue Wang, Shuping Tan, Meihong Xiu, Xiangyang Zhang

**Affiliations:** 1 Department of Neurology, Xuan Wu Hospital of Capital Medical University, Beijing, China; 2 Evidence-based Department, Xuan Wu Hospital of Capital Medical University, Beijing, China; 3 Peking University Huilongguan Clinical Medical School, Beijing Huilongguan Hospital, Beijing, China; 4 CAS Key Laboratory of Mental Health, Institute of Psychology, Chinese Academy of Sciences, Beijing, China

## Abstract

This cohort study compares the psychological status of Chinese adolescents at school before the COVID-19 pandemic and at home during the pandemic to assess whether school attendance was associated with negative mental health outcomes.

## Introduction

Chinese schools focus on examination-centered pedagogy, with high levels of academic stress among adolescents.^1^ Investigation of the association with adverse mental status is lacking. From February to April 2020, all Chinese adolescents attended online classes at home during the COVID-19 pandemic lockdown. We compared the psychological status of adolescents at school and at home using baseline psychological data of adolescents while they were studying in school. We hypothesized that school attendance was associated with negative mental health outcomes.

## Methods

In this cohort study, we surveyed 5 representative middle schools in China with similar shutdowns, policies, and levels of circulating virus. We adopted an overall sampling method and conducted 2 rounds of surveys of all students from November 22, 2019, to January 4, 2020 (round 1) and from March 21 to 31, 2020, during lockdown (round 2). All adolescents were in grades 7 (aged 13 years), 8 (aged 14 years), and 9 (aged 15 years). The protocol was approved by the Ethics in Human Research Committee of the Third Affiliated Hospital of Beijing University of Chinese Medicine. Written informed consent was obtained from parents and guardians. This study followed the STROBE reporting guideline.

Depression and anxiety symptoms were measured using the 9-item Patient Health Questionnaire (range, 0-27, with higher scores indicating more symptoms) and 7-item Generalized Anxiety Disorder Scale (GAD-7; range, 0-21, with higher scores indicating more symptoms).^2^ Cutoff scores were set at 10. For statistical analysis, we used SAS software, version 9.2 (SAS Institute Inc), with significance set at 2-sided *P* = .05. Data were analyzed from April 17 to July 18, 2021. We adjusted the effect of nonresponse using the ratio and regression estimation.

## Results

Respondents returned 13 637 valid questionnaires in round 1 and 10 216 in round 2. Adolescents were older in round 2 (mean [SD] age, 14.33 [1.12] vs 13.77 [1.02] years) (*P* < .001). There was no difference in sex distribution between round 1 (51.28% boys and 48.72% girls) vs round 2 (51.11% boys and 48.89% girls) (*P* = .79) (Table). The adolescents in round 1 reported a higher rate of depression (odds ratio, 1.36 [95% CI, 1.30-1.42]; *P* < .001) and anxiety (odds ratio, 1.61 [95% CI,1.51-1.71]; *P* < .001) symptoms compared with round 2.

**Table.** Demographic and Psychological Characteristics of Respondents in Rounds 1 and 2^a^

Characteristic	School environment (round 1 [n = 13 637])	Home environment (round 2 [n = 10 216])	*P* value	Result (95% CI)
**Demographic**				
Age, mean (SD)	13.77 (1.02)	14.33 (1.12)	<.001	−0.56 (−0.58 to −0.53)^b^
Sex, No. (%)				
Boys	6993 (51.28)	5221 (51.11)	.79	1.00 (0.97 to 1.03)^c^
Girls	6644 (48.72)	4995 (48.89)		
**Psychological**				
PHQ-9 score, median (IQR)	5 (2-9)	3 (0-7)	<.001	1.66 (1.52 to 1.81)^b^
Depression symptom score, No. (%)				
0-9	10 591 (77.66)	8698 (85.14)	<.001	1.36 (1.30 to 1.42)^c^
10-27	3046 (22.34)	1518 (14.86)		
Depression symptom category (score), No. (%)				
Normal (0-4)	6612 (48.49)	6304 (61.71)	<.001	NA
Mild (5-9)	3979 (29.18)	2394 (23.43)		
Moderate (10-14)	1685 (12.36)	878 (8.59)		
Severe (15-27)	1361 (9.98)	640 (6.26)		
GAD-7 score, median (IQR)	3 (1-7)	1 (0-4)	<.001	1.77 (1.65 to 1.89)^b^
Anxiety symptom score, No. (%)				
0-9	11 670 (85.58)	9456 (92.56)	<.001	1.61 (1.51 to 1.71)^c^
10-21	1967 (14.42)	760 (7.44)		
Anxiety symptom category (score), No. (%)				
Normal (0-4)	8382 (61.47)	7792 (76.27)	<.001	NA
Mild (5-9)	3288 (24.11)	1664 (16.29)		
Moderate (10-14)	1217 (8.92)	438 (4.29)		
Severe (15-21)	750 (5.50)	322 (3.15)		

Abbreviations: GAD-7, 7-item Generalized Anxiety Disorder; NA, not applicable; OR, odds ratio; PHQ-9, 9-item Patient Health Questionnaire.

^a^ Percentages have been rounded and may not total 100.

^b^ Values are expressed as mean difference.

^c^ Values are expressed as odds ratio.

We randomly selected 202 nonrespondents in round 2 and ensured they could complete the questionnaire online. No significant nonrespondent bias was found in round 2. In round 2, rates were 14.86% (95% CI, 14.17%-15.55%) for depression and 7.44% (95% CI, 6.93%-7.95%) for anxiety. After adjusting for the effect of nonresponse, the rates were 14.26% (95% CI, 13.60%-14.92%) and 8.28% (95% CI, 7.71%-8.85%), respectively.

## Discussion

Chinese education focuses on obtaining high scores in College Entrance Examination through repeated examinations, and academic stress may cause depression and anxiety.^3,4^ To obtain better academic performance, 63.9% of Chinese adolescents sacrifice sleep and attend extracurricular classes, and 51.0% of adolescents do not get optimal sleep.^1^ Sleep deprivation among adolescents may also contribute to anxiety and depression.^5^ Social interactions (eg, social anxiety) and peer-related stressors may also play roles in mental health. Thus, after lockdown, the removal from school and extracurricular activities and more sleep and access to peers via social media may help reduce adolescents’ stress.

However, some factors of lockdown may be associated with worsened mental health, such as disengagement from face-to-face peer contact and potential exposure to parental distress, financial pressure, and domestic violence. Although we did not consider these variables, the prevalence of depression and anxiety at round 2 was lower than at round 1. The psychological impact of schooling on adolescents may be greater than that of these variables.

The findings of this cohort study suggest that the school environment is associated with psychological stress, alleviated after leaving school. Our study was limited by using a pen-paper survey in round 1 and online survey in round 2. In addition, owing to pandemic limitations, we could not ensure that all adolescents in round 1 responded to the round 2 survey.
